# Inflammatory bowel disease (IBD) in horses: a retrospective study exploring the value of different diagnostic approaches

**DOI:** 10.1186/s12917-018-1343-1

**Published:** 2018-01-19

**Authors:** Berit Boshuizen, Margreet Ploeg, Jeroen Dewulf, Sanne Klooster, Marco de Bruijn, Marie- Thérèse Picavet, Katrien Palmers, Lukas Plancke, Hilde De Cock, Mathijs Theelen, Catherine Delesalle

**Affiliations:** 1 Department of Virology, Parasitology and Immunology, Research Group of Comparative Physiology, Faculty of Veterinary Medicine, Ghent University, Merelbeke, Belgium; 2 Wolvega Equine Clinic, Oldeholtpade, Wolvega, The Netherlands; 3 Department of Pathobiology, Faculty of Veterinary Medicine, Utrecht University, Utrecht, The Netherlands; 4 Department of Obstetrics, Reproduction and Herd health, Faculty of Veterinary Medicine, Ghent University, Merelbeke, Belgium; 5 Department of Equine Sciences, Faculty of Veterinary Medicine, Utrecht University, Utrecht, The Netherlands; 6 DBP Veterinary Services, Moerbeke-Waas, Belgium; 7 De Morette Equine Clinic, Asse, Belgium; 8 Veterinary Pathology Services/Medvet laboratory, Antwerp, Belgium

**Keywords:** Equine, Chronic enteritis, Weight loss, Colic, Duodenal biopsy, Oral glucose tolerance test

## Abstract

**Background:**

Diagnosing IBD in horses is challenging and requires a multimodal approach, since no conclusive diagnostic test is available.

The objectives of this study were to provide an overview of population characteristics, results of applied diagnostic tests, treatment modalities and outcome in a large group of horses thought to have IBD and that were presented to four large equine referral hospitals, and to provide an exploratory investigation of possible associations between results of applied diagnostic tests, applied treatment modalities and outcome.

A retrospective case series was performed across four large equine referral hospitals. Seventy-eight horses, thought to have IBD were included. Case history, clinical findings, diagnostic test results including oral glucose tolerance test (OGTT) and enteral biopsies (both duodenal and rectal), applied therapy and outcome were studied. A Chi-Square test was used to identify associations between results of diagnostic tests, treatment and outcome. *P*-values < 0.05 were considered significant.

**Results:**

Lethargy, diarrhoea, recurrent colic and weight loss were recorded in respectively 21,8%; 14,1%; 28,2% and 78,2% of cases. Over 70% of horses thought to have IBD had an abnormal OGTT. Only weight loss was significantly associated with aberrant enteral biopsy results, but not with abnormal OGTT results or low blood total protein. There was no association between an aberrant biopsy result and a disturbed OGTT. There was no association between either OGTT results or enteral biopsy results and a low blood total protein content, presence of gastric ulcer disease or an aberrant endoscopic aspect of the duodenal entrance.

**Conclusions:**

Weight loss is a highly prevalent symptom in IBD suspected horses. Enteral biopsies may be a useful diagnostic aid in the work-up of horses thought to suffer from IBD, however further research is required to demonstrate their true diagnostic value. Until more standardized scientific research is available, one should be careful with the interpretation of enteral biopsy results There is a need for better standardization of enteral biopsy procedures and the histopathological scoring of biopsies.

**Electronic supplementary material:**

The online version of this article (10.1186/s12917-018-1343-1) contains supplementary material, which is available to authorized users.

## Background

Equine inflammatory bowel disease (IBD) is a challenge to diagnose, adequately treat and manage. Depending on the main type of inflammatory cells involved, several different types of IBD are histopathologically distinguished: granulomatous enteritis (GE), multisystemic eosinophilic epitheliotropic disease (MEED), lymphocytic-plasmacytic enterocolitis (LPE), diffuse eosinophilic enterocolitis (DEE) and proliferative enteritis (PE), each of which can affect either small and/or large intestine [1–3]. Most horses present with problems such as recurrent colic, weight loss often despite good appetite, poor performance, lethargy, ventral oedema and they can suffer from hypoproteinaemia and hypoalbuminaemia [1–6]. Prognosis is reported to be guarded [2, 5, 6].

Definitive confirmation of IBD diagnosis can only be obtained by histopathological assessment of intestinal full thickness biopsies harvested during either exploratory laparotomy or laparoscopy or post-mortem excisions during autopsy [4, 5]. Due to the lack of a distinctive non-invasive diagnostic test, diagnosis of IBD requires a multimodal approach and only leads to a presumptive diagnosis. The most commonly used non-invasive diagnostic tool is the oral glucose tolerance test (OGTT) to assess malabsorption, of which IBD is the major cause [5, 7]. Specificity and sensitivity are reported to be respectively 90–100% and 40–45% for total malabsorption [5, 8, 17]. The diagnostic value of other non-invasive diagnostic approaches has been explored to a much lesser extent. Duodenal biopsies, collected through the biopsy canal of a gastroduodenoscope, have become a quite common diagnostic approach, in addition to the collection of rectal biopsies. [1, 3, 4, 9–15]. Duodenal and rectal biopsies seem a very elegant diagnostic aid to determine the patients’ prognosis. They are technically quite easy to obtain and not harmful [22, 16]. However, it is not known whether histopathological evaluation of these biopsies is correlated to other tests routinely applied in the work-up of horses thought to have IBD. To obtain a better view on this issue, the following study was set up, as a joint venture between the Utrecht University Equine Clinic (The Netherlands) (referral centre 1), The Wolvega Equine Clinic (The Netherlands) (referral centre 2), The Morette Equine Clinic (Belgium) (referral centre 3) and The Bosdreef Equine Clinic (Belgium) (referral centre 4). The objectives of this retrospective study were: (1) to provide an overview of patient data, diagnostic workup and outcome of a large group of horses suspected to have IBD, and (2) to study possible associations between results of applied diagnostic tests, applied treatment modalities and outcome. To our knowledge, this is the first large-scale study with a multicentre approach to study the diagnostic work-up of horses with IBD [1, 2, 4, 17–20].

## Methods

### Study population characteristics

The study population encompassed 83 horses suspected of suffering from IBD that were admitted to one of four involved referral centres over the course of two years. Inclusion criteria were 1) horses suspected of having IBD and 2) subjected to an OGTT in the referral centre. Horses with malignancies (*n* = 5) were excluded. For 78 horses history and case features such as age, breed, gender, month of admission and type of pre-diagnostic diet were retrieved from patient records. For descriptive statistics, signs were categorized as either ‘lethargy/poor performance’, ‘diarrhoea’, ‘recurrent colic’ or ‘weight loss’ [1–4].

### Routine diagnostic tests

In most cases, diagnostic tests were performed in following order, starting from minimally invasive towards invasive.

**Serum chemistry** and **hematology **included packed cell volume (PCV), leucocyte count, total protein (TP), albumin, gamma globulins and gamma-glutamyl transferase (GGT). An overview of applied reference ranges per participating referral centre is provided in Table 1.

**Table 1:** Applied reference ranges for tested complete blood count (CBC) and serum chemistry parameters per referral centre

	referral centre 1	referral centre 2	referral centre 3 and 4	% of study population out of reference
Total Protein	52–79 g/L	62–94 g/L	52–67 g/L	11.6% ↓
Albumin	26–37 g/L	23–35 g/L	25–39 g/L	8.7% ↓
Gamma globulin	6–19 g/L	4,6–13,4 g/L	7,9–17,9 g/L	4.5% ↑
Gamma-GT	< 20 U/L	16–56 U/L	< 22 U/L	23.0% ↑
PCV	0,32–0,42 L/L	0,32–0,52 L/L	0,30–0,46 L/L	17.9% ↓
Leucocytes	7,0–10,0 10^9/L	5,5–12,5 10^9/L	5,0–10,0 10^9/L	23.9% ↑

Findings recorded for **rectal examination **were categorized as either: ‘presence of small intestinal loops with increased wall thickness’, ‘presence of large intestinal segments with increased wall thickness’, ‘other abnormal findings’, ‘no abnormal findings’. In case **transabdominal ultrasound **was performed, reported findings were categorized as either: ‘visualization of small intestinal loops with increased wall thickness (> 3 mm)’, ‘visualization of large intestinal segments with increased wall thickness (*>* 4 mm)’ or ‘no abnormalities recorded’. It was recorded whether faeces were tested for sand accumulation and parasitic egg count.

All horses were subjected to a similar **OGTT** protocol in all four referral centres: after 18–24 h starving, a baseline venous sodium-fluoride blood sample was taken and subsequently, 1 g glucose per kg BW was dissolved in 1 L lukewarm water and was given by nasogastric intubation. Then every 30 min venous blood was collected at the jugular vein for four consecutive times, followed by four blood samples taken every hour. The resulting blood glucose curve was deemed normal when a peak glucose value greater than 85% increase over the resting values was reached within 90–120 min and returned to baseline within 6 h. Partial malabsorption was defined as OGTT peak glucose levels reaching 15–85% increase over baseline and total malabsorption when levels remained ≤ 15% increase over baseline [3, 16].

**Gastroduodenoscopic** findings were categorised as either: ‘presence of equine gastric ulcer syndrome (grade 1 or above)’, ‘aberrant aspect of duodenal entrance (hyperaemia, oedema or striations)’ or ‘no aberrant findings’.

### Enteral biopsies

The approach for collection of duodenal biopsies was similar in all 4 referral centres. In the sedated horse, at least two **duodenal biopsies **were harvested using a ± 2.8 mm diameter biopsy catheter with forceps, inserted through the biopsy canal of a gastroduodenoscope [1, 22]. **Rectal biopsies** were only harvested in referral centre 4 and these were collected using an Olympus Japan, K47062 endoscope, equipped with a 4 mm diameter biopsy catheter with forceps that was introduced into the biopsy canal. Horses were restrained in stocks, in most cases without sedation and 2–4 pieces of rectal mucosal tissue were taken in the left upper quadrant of the rectum. All biopsies (duodenal and rectal) were immediately immersed in 10% neutral buffered formalin and sent to the pathology lab (Department of Pathobiology Utrecht University for referral centre 1 and 2 and Medvet Antwerp for referral centre 3 and 4), where they were paraffin-embedded, sectioned at 4.0 μm, and Hematoxylin and eosin (H&E) staining was performed [4, 10, 14, 21, 22].

The reported quality score of the biopsy tissue by the different laboratories was categorized as either: ‘useful diagnostic material’, ‘less than ideal’ (either too small, solely mucosal tissue and/or presence of pinch artefacts), or ‘insufficient to establish a clear diagnosis’. The enteral layers that could be evaluated histopathologically were categorized as either: ‘only mucosa or mostly mucosa’, ‘mucosa and submucosa’ or ‘obscure’.

The recorded enteral biopsy results were categorized as either ‘aberrant’ or ‘normal’ and the assigned IBD type, was categorized as either: ‘granulomatous enteritis’, ‘eosinophilic enteritis, ‘lymphocytic-plasmacytic enteritis’ or ‘mixed enteritis’ for duodenal biopsies and ‘lymphocytic proctitis’, ‘eosinophilic proctitis’ and ‘neutrophilic proctitis’ for rectal biopsies.

### Applied medical therapies and dietary adaptations

The applied medical therapy was categorized as either ‘corticosteroids’ (which covers the use of either prednisolone (1 mg/kg BW po q24h) or dexamethasone (0,06 mg/kg BW po q24h)) or ‘omeprazole’ (2–4 mg/kg BW po q24h). Whether and which dietary adaptations were applied was recorded as well: ‘laxative feeding approach’, ‘increased fibre content in the diet’, ‘low carbohydrate diet’, ‘high fat diet’, ‘high protein diet’, ‘increased pasture turn-out’, ‘bloating preventive diet’, ‘supplementation with probiotics’.

### Long-term follow-up

When horses were admitted multiple times to the referral centre, their revisit was categorized as either: ‘control visit’, ‘visit due to recurrent problems’, ‘colic surgery’ or ‘euthanasia’. A telephone follow-up was performed at least one year after diagnosis. Horse owners were interviewed about the applied dietary management since diagnosis, applied medical therapy and performance of their horse. Outcome was categorized as either ‘performing to expectation’, ‘no amelioration despite therapy’, ‘euthanasia or natural death due to recurrent GI related problems’, ‘euthanasia or natural death due to other causes’.

### Statistical analyses

The following groups were created: age, breed, gender, month of admission, pre-diagnostic diet, clinical signs, OGTT, blood work, rectal palpation, transabdominal ultrasound, gastroduodenoscopy, duodenal biopsy, rectal biopsy, applied therapy and outcome. For purposes of descriptive statistics, for each group, results were categorized as mentioned above. A detailed overview of these descriptive data can be found in Additional file 1: Figure S1, Additional file 2: Figure S2 and Additional file 3: Figure S3. For continuous outcomes, results are presented as mean ± standard deviation (SD). For categorical data, frequencies of occurrence are presented. Since only three horses showed total malabsorption, it was decided to merge these horses with the partial malabsorption group throughout all statistical analyses. All categorical variables were made binary for statistical analysis. For enteral biopsy results and gastroduodenoscopic findings the created categories for descriptive statistics were dichotomized as either ‘aberrant’ or ‘normal’. All pre-diagnostic diet options were classified into two groups: either ‘pelleted diet’ or ‘no pellets’. Blood results were categorized as either ‘within’ or ‘outside’ reference range. For rectal palpation and transabdominal ultrasound the findings were categorized as either ‘small intestinal loops with increased wall thickness’ or ‘absence of small intestinal loops with increased wall thickness’. Also medical therapy, dietary changes, and long term follow up (negative or positive outcome) were made binary. Possible associations between results of applied diagnostic tests, applied treatment modalities and outcome were studied by means of Chi-Square analyses, SPSS (version 22.0). *P*-values < 0.05 were considered significant.

## Results

### Study population characteristics

The study population encompassed warmblood horses (*n* = 60), Standardbreds (*n* = 4), Friesian horses (*n* = 8) and ponies (n = 6). Geldings (47.4%) were most commonly represented followed by mares (35.9%) and stallions (16.7%). There was no gender predisposition (*p* = 0.55), nor seasonality (*p* = 0.37) in the pattern of admittance. The average age at admission was 8.95 ± 4.9 years (range 1–25 years).

Of 47 horses, the dietary management prior to referral was recorded: hay (68.1%), haylage (19.1%), combination of hay and haylage (6.4%), pelleted feed (74.5%), muesli (31.9%), oats (4.3%) and alfalfa (4.3%). The majority of horses had access to pasture turnout (66.7%) and had pellets in their diet (74.5%). A combination of pellets and muesli was fed in 10.6% of cases. There was no association between pellets in the diet and either a disturbed OGTT (*p* = 0.17), aberrant gastroduodenoscopic findings (*p* = 0.69) or an aberrant duodenal (*p* = 0.89) or rectal biopsy result (*p* = 0.42).

### Clinical signs

‘Weight loss’, ‘recurrent colic’, ‘lethargy’ and ‘diarrhoea’ were recorded in respectively 78.2%, 28.2%, 21.8% and 14.1% of cases. ‘Weight loss’ was recorded in 61 horses, the majority of which showed ‘weight loss despite good appetite’ (41 horses, 67.2%). None of these signs were significantly associated with abnormal OGTT results.

### Diagnostic test results other than enteral biopsies

Rectal palpation was performed in 72 (92.3%) horses and in a minority of cases (22 horses (30.6%)) thick-walled small intestinal loops were identified. Of these 22 horses, 19 (86.4%) had an aberrant OGTT result, but also from 50 horses where no thickened small intestinal loops were identified 34 (68%) horses had abnormal OGTT results. No association could be identified between these findings and aberrant OGTT results (*p* = 0.09).

No association was found between identifying thick walled small intestinal loops during rectal palpation and an aberrant duodenal biopsy result (*p* = 0.49). Additional file 1: Figure S1 gives a detailed overview of the study population, which is depicted as a flow chart, starting with two groups (normal and abnormal OGTT) and depicting for each group the obtained results/findings for respectively duodenal biopsies, rectal biopsies and rectal palpation.

Thick walled large intestinal segments were identified in only 7 cases (9.7%) during rectal palpation. From 5 of these cases a rectal biopsy result was available and categorized as aberrant in all 5 cases.

Faeces were tested for sand accumulation in 44 cases and parasitic egg count was performed in 62 cases. In 4 horses (9.1%) a faecal accumulation of sand was reported and 10 horses (16.1%) had a positive parasitic egg count for Strongylus type eggs (> 150 EPG).

Transabdominal ultrasound was performed in 57 horses (73%). Additional file 2: Figure S2 provides a detailed overview of the study population, which is depicted as a flow chart, starting with two groups (normal and abnormal OGTT) and depicting for each group the obtained results/findings for respectively gastroduodenoscopy, transabdominal ultrasound and rectal palpation.

Ultrasound revealed thick walled small intestinal loops in 16 cases and thick walled large intestinal segments in 9 cases. In 72.2% (*n* = 39) of cases with an aberrant OGTT result, transabdominal ultrasound was performed and 33.3% (*n* = 13) revealed presence of thick-walled small intestinal loops. There was a significant association between finding thickened small intestinal loops during rectal palpation and visualizing them by means of transabdominal ultrasound (81.8%; *p* < 0.01). No association was found between visualization of thick walled small intestinal loops during transabdominal ultrasound and either a disturbed OGTT (*p* = 0.19), a low blood TP (*p* = 0.80) or an aberrant duodenal biopsy result (*p* = 0.29).

Serum chemistries and CBCs were available for 69 cases. Additional file 3: Figure S3 provides an overview of the bloodwork of the horses included in this study. The data is divided in normal and abnormal OGTT results.

The majority of horses suspected of suffering from IBD (70.5%, *n* = 55 horses) had an abnormal OGTT result (Additional file 1: Figure S1, Additional file 2: Figure S2 and Additional file 3: Figure S3) and in 33 of these horses (60%) an aberrant duodenal (*n* = 15) or rectal biopsy (*n* = 21) was identified. There was no association between a disturbed OGTT and presence of thick-walled small intestinal loops on either rectal palpation or transabdominal ultrasound (*p* = 0.55 and *p* = 0.70), a low total protein content in the blood (*p* = 0.60), aberrant duodenal (*p* = 0.48) or rectal biopsies (*p* = 0.52) or negative outcome (*p* = 0.94). There was no significant gender predisposition for a disturbed OGTT (p = 0.55).

Gastroduodenoscopy was performed in 60 horses (Additional file 2: Figure S2). In 5 horses (8.3%) an abnormal aspect of the duodenum was found, and in two of these cases signs of EGUS were also found. Nearly half of the horses with an abnormal OGTT result (*n* = 42) showed abnormalities (EGUS or abnormal aspect of the entrance of the duodenum) during gastroduodenoscopy (47.6%, *n* = 20, *p* = 0.30). Likewise, nearly half (41.4%, *n* = 24, *p* = 0.45) of the horses with an aberrant enteral biopsy result showed abnormalities during gastroduodenoscopy. All horses with an aberrant aspect of the duodenum during gastroduodenoscopy (*n* = 5) also had an abnormal OGTT result, but only 2 of these cases showed an aberrant enteral biopsy.

### Enteral biopsies

Discomfort was rarely shown during the procedure and no complications were encountered. On all occasions enteral biopsies were deemed of moderate or lower quality (Table 2). Rectal biopsies were of moderate quality in 93.3% of cases whereas duodenal biopsies were of moderate quality in only 56.8% of cases. Quality assessment of duodenal biopsies differed significantly between clinics: biopsies taken at referral centre 1 and 2 were of useful quality in 50% of cases, whereas 76.9% of duodenal biopsies taken at referral centre 3 and 4 were labelled as useful. The majority (86.5%) of the duodenal biopsies consisted only of intestinal mucosal tissue. Rectal biopsies were in general more profound: 64.4% consisted of only mucosa or mostly mucosa, 31.1% consisted of both mucosa and submucosa and 4.5% were labelled as obscure.

**Table 2:** Detailed overview of the quality assessment of enteral biopsies across involved pathology labs

	Duodenal biopsies	Rectal biopsies
Total number of biopsies	100% (*n* = 37)	100% (*n* = 45)
Good quality diagnostic material	0% (*n* = 0)	0% (n = 0)
Moderate quality	56.8% (n = 21)	93.3% (n = 42)
Poor quality	18.9% (*n* = 7)	0% (n = 0)
Insufficient quality to assess	24,3% (n = 9)	6,7% (n = 3)

In 37 horses (47.4%) a duodenal biopsy was collected; 8 of these horses had a normal OGTT and 29 of these horses had an abnormal OGTT (Additional file 1: Figure S1). The duodenal biopsies showed histopathological features of inflammation in 56.8% of the cases. In 45 horses (57.7%) a rectal biopsy was harvested. The majority of rectal biopsies (84.4%) showed inflammatory features. In 10 out of 78 horses (12.8%) both duodenal and rectal biopsies were harvested. In 7 of these horses both biopsies were aberrant showing the same inflammatory patterns in duodenal and rectal biopsies and only 3 of these horses (42.9%) showed abnormal OGTT result.

For horses with a normal glucose response, only in a limited number of cases duodenal biopsies were collected, however 75% (6 out of 8 horses) showed aberrant histopathological results. Rectal biopsies were taken more frequently in case of a normal OGTT result, with an aberrant result in 84,2% of 19 cases. Specified biopsy results can be found in Table 3. A biopsy can fall into more than one category in this table, for instance when a mixed enteritis was found consisting of both eosinophilic and lymphocytic plasmacytic components.

**Table 3:** Detailed overview of the histopathological classification of the enteral biopsies

	OGTT > 85% (normal)		OGTT < 85% (abnormal)	
	Duodenum	Rectum	Duodenum	Rectum
Normal	1	4	4	1
Granulomatous enteritis	0	0	4	0
Eosinophilic enteritis	1	9	2	13
Lymphocytic plasmacytic enteritis or proctitis	5	15	11	21
Neutrophilic inflammation	0	1	0	2

Aberrant duodenal and rectal biopsy results were both significantly associated with weight loss (duodenal: *p* = 0.03, rectal: *p* = 0.04).

There was no association found between either duodenal biopsy or rectal biopsy test results or blood test results: aberrant white blood cell count (duodenal *p* = 0.25 and rectal biopsy *p* = 0.68), PCV (*p* = 0.36 and *p* = 0.60), TP (*p* = 0.31 and *p* = 0.39), albumin (*p* = 0.44 and *p* = 0.50), gamma globulin (*p* = 0.41 and p = 0.50) or GGT (*p* = 0.91 and *p* = 0.10).

### Dietary management and treatment

A diet recommendation was provided in 62.8% (*n* = 49) of the horses. In most cases it concerned horses with an aberrant OGTT result. In 15 horses a dietary advice was provided despite a normal OGTT test result. The different kinds of diets that were advised were either ‘laxative’ (*n* = 8: 16.3%), ‘high-protein’ (*n* = 18: 36.7%), high-fat’ (*n* = 31: 63.3%), ‘high-fibre’ (*n* = 40: 81.6%), ‘pasture turn out’ (*n* = 15: 30.6%) and ‘probiotics’ (*n* = 10: 20.4%). Dietary changes aiming for an increase in total fat content (63.3%) and fibre content (81,6%) were advised most often. There was no association between dietary changes and positive outcome (*p* = 0.75).

Medical treatment was applied in 68 horses. An overview of the applied treatments is provided in Additional file 4: Figure S4. Prednisolone was administered most often.

No association between applied treatments and positive outcome could be found (prednisolone *p* = 0.17, dexamethasone *p* = 0.65 and omeprazole *p* = 0.73).

### Follow up

Long-term follow up was achieved in 39 horses. Only a minority of cases was presented to the clinic for a control visit (*n* = 17) or for recurrence of problems (*n* = 9, of which 4 horses were euthanized shortly after admission). A telephone questionnaire retrieved additional information. In total, 12 out of 39 (30,8%) horses died, 10 of which were euthanized because of suffering from IBD and 2 due to orthopaedic problems. From these 10 horses, 8 horses showed an abnormal OGTT. In 4 horses a rectal biopsy was available (all aberrant) and in 4 horses a duodenal biopsy was available (3 aberrant, 1 of insufficient quality). None of the euthanized horses showed gross pathological signs of malignancy during autopsy.

## Discussion

The current retrospective case series provides an overview of population characteristics, results of applied diagnostic tests, applied treatment modalities and outcome in a large group of horses suspected of suffering from IBD and presented to four equine referral hospitals. Special attention was given to duodenal biopsies, which are commonly included in IBD diagnostic work-up. However, very little is known about their diagnostic value.

### Study population characteristics

Recurrent colic and weight loss despite good appetite are signs that are often reported in equine IBD [2–4, 10, 23]. The results of our study are in accordance with this. Aberrant duodenal and rectal biopsy results were both significantly associated with weight loss.

Horses were predominantly admitted during their active sports career and no gender predisposition could be identified. Ulcerative colitis, a subtype of human IBD, is diagnosed with a higher incidence in female patients [24, 25]. Human IBD is caused by a combination of familial predisposition and certain dietary habits [26, 27], thus the equine diet could be an interesting factor to focus on. However, in this study no significant association was found between pre-diagnostic feeding management and a disturbed OGTT or a disturbed enteral biopsy result. The possible link between feeding management and IBD has been suggested in several equine studies, however, no study has been able to pinpoint this [18, 21, 28]. A possible explanation for this is that equine IBD manifests itself in many forms and the heterogeneity of the studied populations results in poor statistical power.

### Response to therapy and prognosis

Medical treatment was not associated with positive long-term outcome in this study. However, only 68 horses received medical treatment, of which 56 were treated with prednisolone (see Additional file 4). Prednisolone was used most often for IBD patients in the four referral centres. The inability to find a positive effect of prednisolone on long-term outcome could be erroneous due to a type II statistical error, since for only 31 of these prednisolone treated horses a follow-up was available. However, 19 of these 31 (61,3%) prednisolone-treated horses were categorized by means of the telephone questionnaire as performing to expectation, which is in accordance with Kaikkonen [10].

Hypoproteinaemia and hypoalbuminemia were not highly prevalent and overall prognosis was reasonably good for the patients in our study: only 10 out of 39 horses for which long term follow-up was available, did not survive after one year because of IBD related complaints, which is not in accordance with previous studies. [4, 5, 10] Kaikkonen et al. used hypoproteinaemia and hypoalbuminemia as essential inclusion criteria, thereby possibly excluding early, or milder IBD cases [10]. Up until now, most equine IBD related studies included post mortem histopathological assessment of full thickness enteral specimens collected during necropsy as definitive diagnosis and Kemper only focused on the lymfoplasmacytic enterocolitis subtype [4, 5]. In our retrospective study full-thickness biopsies were not available, which is a limitation. However, the surgical harvest of multiple enteral full thickness biopsies is not practical in horses with IBD, both from a benefit-risk ratio as from an economical point of view, especially in mild to moderate cases [29].

### Applied diagnostics and their relationship with OGTT and enteral biopsy results

A significant association between finding thickened small intestinal loops on rectal palpation and visualizing them during abdominal ultrasound was found, with ultrasound being more sensitive. However, finding these thick walled small intestinal loops is not significantly associated with a low blood TP content, a disturbed OGTT, or an aberrant enteral biopsy result, which is important to keep in mind during diagnostic work-up.

Another interesting finding is that in all 5 horses in which an abnormal aspect of the duodenal entrance was seen during gastroduodenoscopy, an abnormal OGTT was found, but only 2 out of 5 horses also showed aberrant duodenal biopsies. This finding needs further study in a larger number of horses. It could suggest that a biopsy of the entrance of the duodenum is not sufficiently representative for the more distal small intestine, of which the total absorptive capacity is reflected in OGTT results. In that case, more research should be directed towards for example development of elegant laparoscopic techniques to harvest full thickness enteral biopsies in horses thought to have IBD. Normal OGTT was shown not to exclude IBD or other infiltrative enteral diseases [16, 30]. Indeed, OGTT takes glucose uptake only into account, which could explain the low reported sensitivity of OGTT (40–45%). In the current case series, only 3 horses suffered from total malabsorption. Therefore, the horses suffering from ‘partial malabsorption’ and ‘total malabsorption’ were merged into the category ‘aberrant OGTT’. Therefore, reported sensitivity (40–45%) and specificity (90–100%) for ‘total malabsorption identified by OGTT’ do not apply to our study population, because the incorporation of horses with partial malabsorption will increase the sensitivity and decrease specificity [5, 16, 8, 7]. Despite the aforementioned flaws associated with OGTT, compared to all applicable test, OGTT is deemed very useful for diagnosing IBD. Indeed, depending on severity of clinical signs shown by IBD suspected patients and depending on economics, veterinary clinicians tend to start from minimally invasive, to invasive diagnostic methods. Therefore, only IBD suspected horses that underwent an OGTT were included in this study. The authors are aware that the xylose absorption test would have been a more accurate method, but in none of the participating referral centres it was applied [5, 31].

### Enteral biopsy quality and histopathological evaluation

There is great need for systematic studies on the harvesting technique and histopathological interpretation of duodenal and rectal biopsies in horses [10, 14, 21], since similar studies boosted the evaluation of intestinal biopsies in humans [32], but also in dogs and cats [14]. Packer collected full thickness jejunal biopsies taken post mortem from adult horses and provided an essential view on the normal equine jejunal inflammatory cell populations [33]. Tossens aimed to determine the diagnostic value of duodenal and rectal biopsies by comparing control horses with horses admitted for recurrent colic, weight loss or diarrhoea. No significant differences between enteral biopsies from 19 sick and 11 control horses were observed, but more data was deemed needed and no OGTT was performed [34].

Based upon our study results, in horses suspected of suffering from IBD most rectal biopsies are aberrant (82,2%), in comparison to 56.8% of duodenal biopsies. Therefore, the ‘rectal’ biopsies seemed particularly useful in the current case series. The small colon of the horse extends to approximately 15 cm from the anus. This brings up the question as to whether use of a 3 m endoscope to go further into the small colon would be of value in the future, since in people, colonic biopsies are generally used as the gold standard, and it may be possible to increase the usage of colonic biopsies (at least of the descending colon) in horses relatively non-invasively.

The clinical implication of certain histological findings of rectal biopsies are not clear so far, e.g. Lindberg et al. [35] found heavy eosinophila in the rectal lamina propria and submucosa in respectively 4% and 23% of 21 healthy horses. To investigate whether rectal and/or duodenal biopsies are the best diagnostic approach, a prospective study involving both rectal and gastroduodenoscopic guided duodenal pinch biopsies, OGTT and surgically harvested full-thickness biopsies should be performed.

We found no association between duodenal biopsy or rectal biopsy test results, blood results, rectal palpation findings or gastroscopy and OGTT results. The disappointing quality of the duodenal biopsies needs future attention, since it is very probable that this has a negative effect on histopathological interpretation. Secondly, the lack of a standardized protocol to interpret these biopsies histopathologically needs future attention, as has been illustrated by the difference in quality assessment reported by the different pathology labs that were engaged by the participating referral centres.

## Conclusions

Enteral biopsies may be a useful diagnostic aid in the work-up of horses thought to have IBD, however further research is required to demonstrate their true diagnostic value. There is need for standardization of the biopsy procedure and of the histopathological interpretation protocol. It is important to combine biopsy results with all other applied diagnostic tests during the work up of a horse thought to have IBD and not to overestimate the diagnostic and prognostic value of any diagnostic test.

## Additional files

Additional file 1: Detailed overview of the classification of the study population by OGTT results, and for enteral biopsy and rectal palpation findings. (PDF 250 kb)

Additional file 2: Detailed overview of gastroduodenoscopic and transabdominal ultrasound findings classified by OGTT results in suspected IBD patients. (PDF 387 kb)

Additional file 3: Detailed overview of serum chemistry and complete blood count results from horses suspected of having IBD classified by OGTT results (normal or abnormal). (PDF 388 kb)

Additional file 4: Detailed overview of applied treatments in the study population. (DOCX 14 kb)

## Abbreviations

IBD: Inflammatory bowel disease
OGTT: Oral glucose tolerance test
GE: Granulomatous enteritis
MEED: Multisystemic eosinophilic epitheliotropic disease
LPE: Lymphocytic-plasmacytic enterocolitis
DEE: diffuse eosinophilic enterocolitis
PE: proliferative enteritis
PCV: Packed cell volume
TP: Total protein
GGT: Gamma-glutamyl transferase
CBC: Complete blood count
H&E: Hematoxylin and eosin staining
GI: Gastrointestinal
EGUS: Equine gastric ulcer syndrome
