# Supplementary material for: Inflammatory bowel disease (IBD) in horses: a retrospective study exploring the value of different diagnostic approaches
Source: BMC Vet Res. 2018 Jan 19;14:21. doi: 10.1186/s12917-018-1343-1 (PMC5775604; doi:10.1186/s12917-018-1343-1)
Supplement: Supplementary file 1 — Detailed overview of the classification of the study population by OGTT results, and for enteral biopsy and rectal palpation findings. (PDF 250 kb) [file 12917_2018_1343_MOESM1_ESM.pdf]

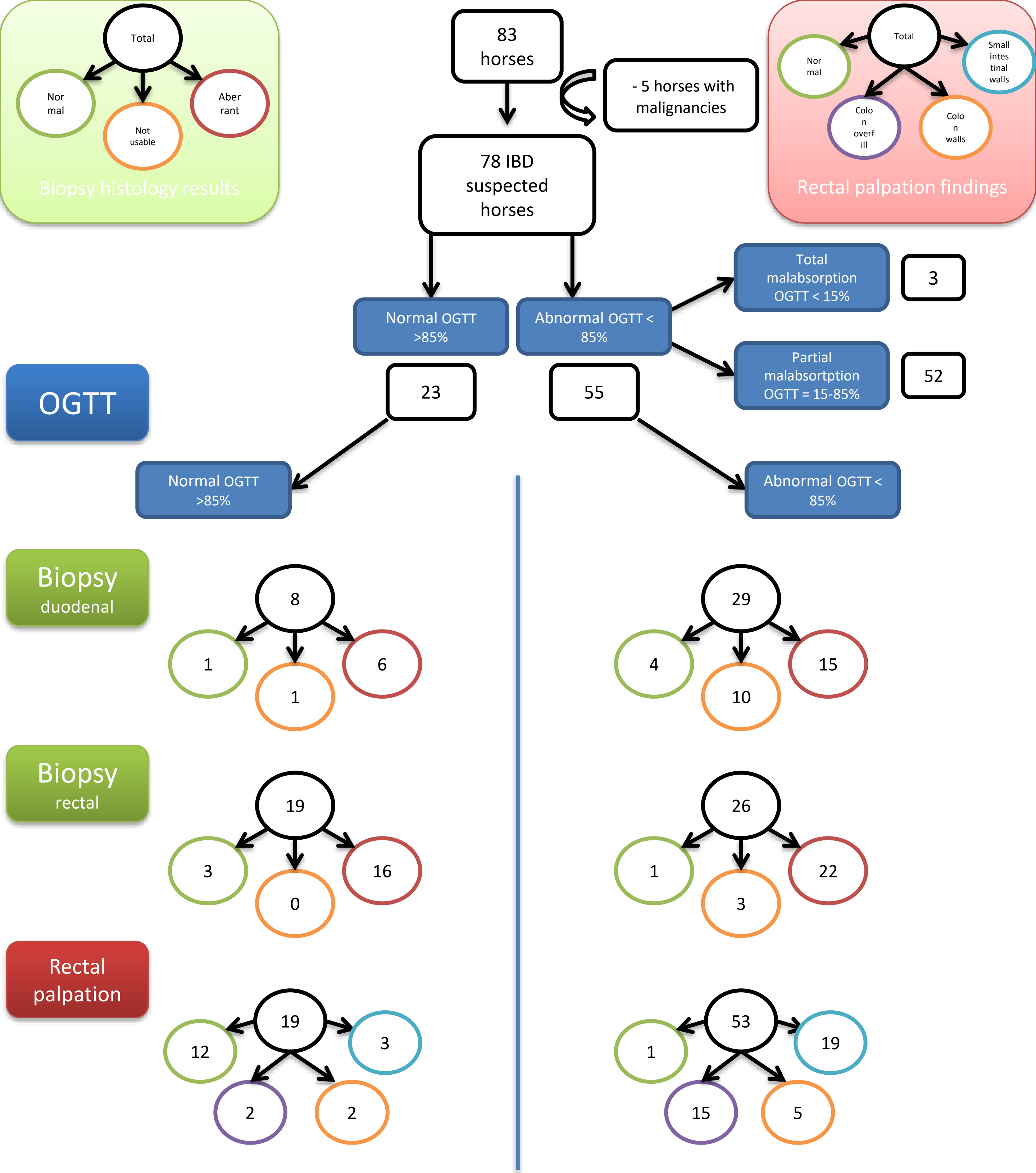

Additional figure 1: Overview of the equine IBD suspected study population, classified by OGTT result (either normal: left panel, or abnormal: right panel), depicting enteral biopsy results (either duodenal (top panel) or rectal (middle panel) and rectal palpation results (bottom panel). In the left and right upper corner colour codes are depicted for classification of respectively enteral biopsy (left panel) and rectal palpation (right panel).
