# Supplementary material for: Inflammatory bowel disease (IBD) in horses: a retrospective study exploring the value of different diagnostic approaches
Source: BMC Vet Res. 2018 Jan 19;14:21. doi: 10.1186/s12917-018-1343-1 (PMC5775604; doi:10.1186/s12917-018-1343-1)
Supplement: Supplementary file 2 — Detailed overview of gastroduodenoscopic and transabdominal ultrasound findings classified by OGTT results in suspected IBD patients. (PDF 387 kb) [file 12917_2018_1343_MOESM2_ESM.pdf]

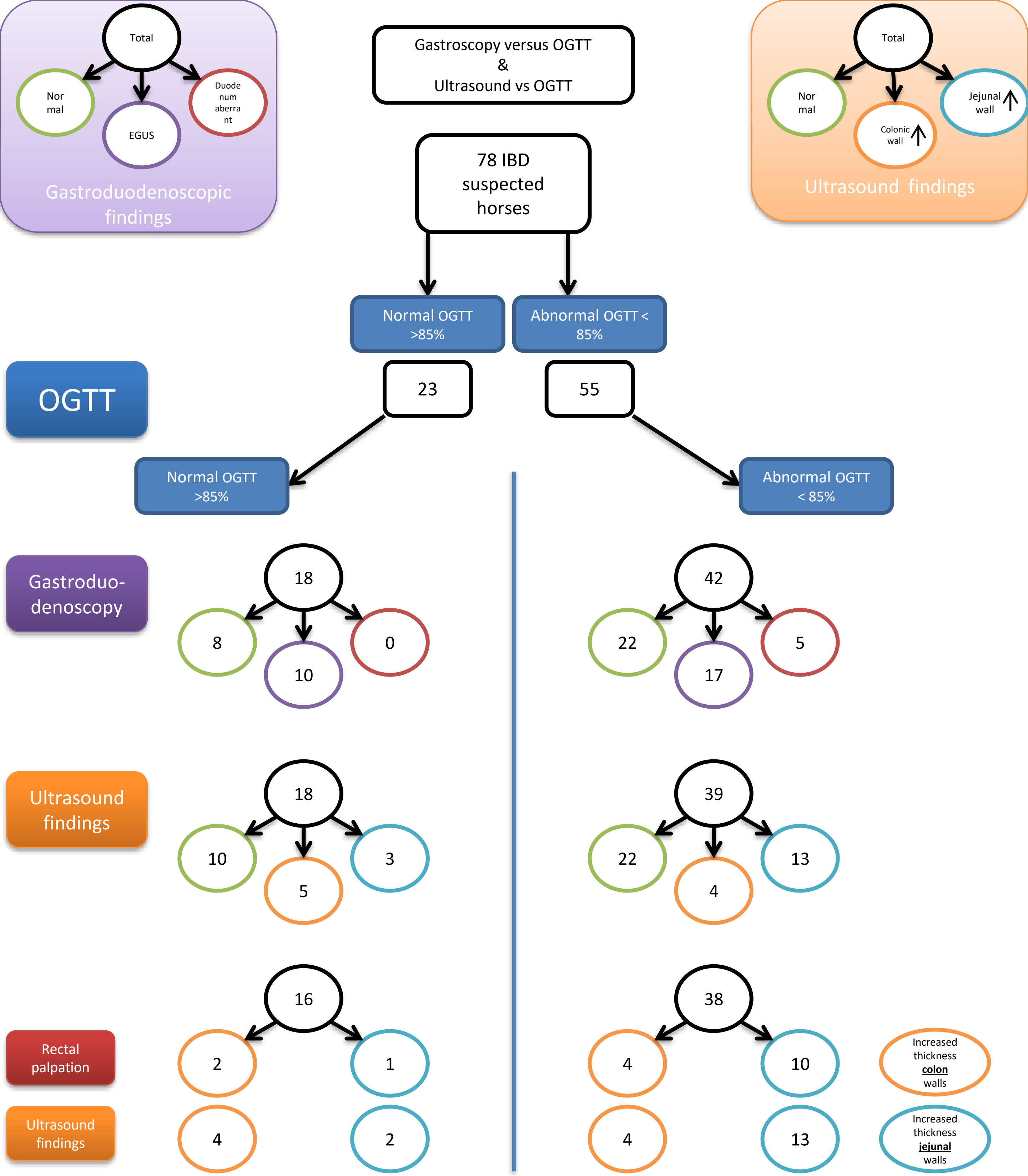

Additional figure 2: Overview of gastroduodenoscopic and transabdominal ultrasound findings within the equine IBD suspected study population, classified by OGTT result (either normal: left panel, or abnormal: right panel). In the left and right upper corner the colour codes are depicted for classification of respectively gastroduodenoscopy (left upper corner) and ultrasound findings (right upper corner). Finding increased enteric wall thickness by rectal palpation or using transabdominal ultrasound are both compared in the bottom panel. Colour codes for the different enteric walls are depicted in the right lower corner.
