# Supplementary material for: Inflammatory bowel disease (IBD) in horses: a retrospective study exploring the value of different diagnostic approaches
Source: BMC Vet Res. 2018 Jan 19;14:21. doi: 10.1186/s12917-018-1343-1 (PMC5775604; doi:10.1186/s12917-018-1343-1)
Supplement: Supplementary file 3 — Detailed overview of serum chemistry and complete blood count results from horses suspected of having IBD classified by OGTT results (normal or abnormal). (PDF 388 kb) [file 12917_2018_1343_MOESM3_ESM.pdf]

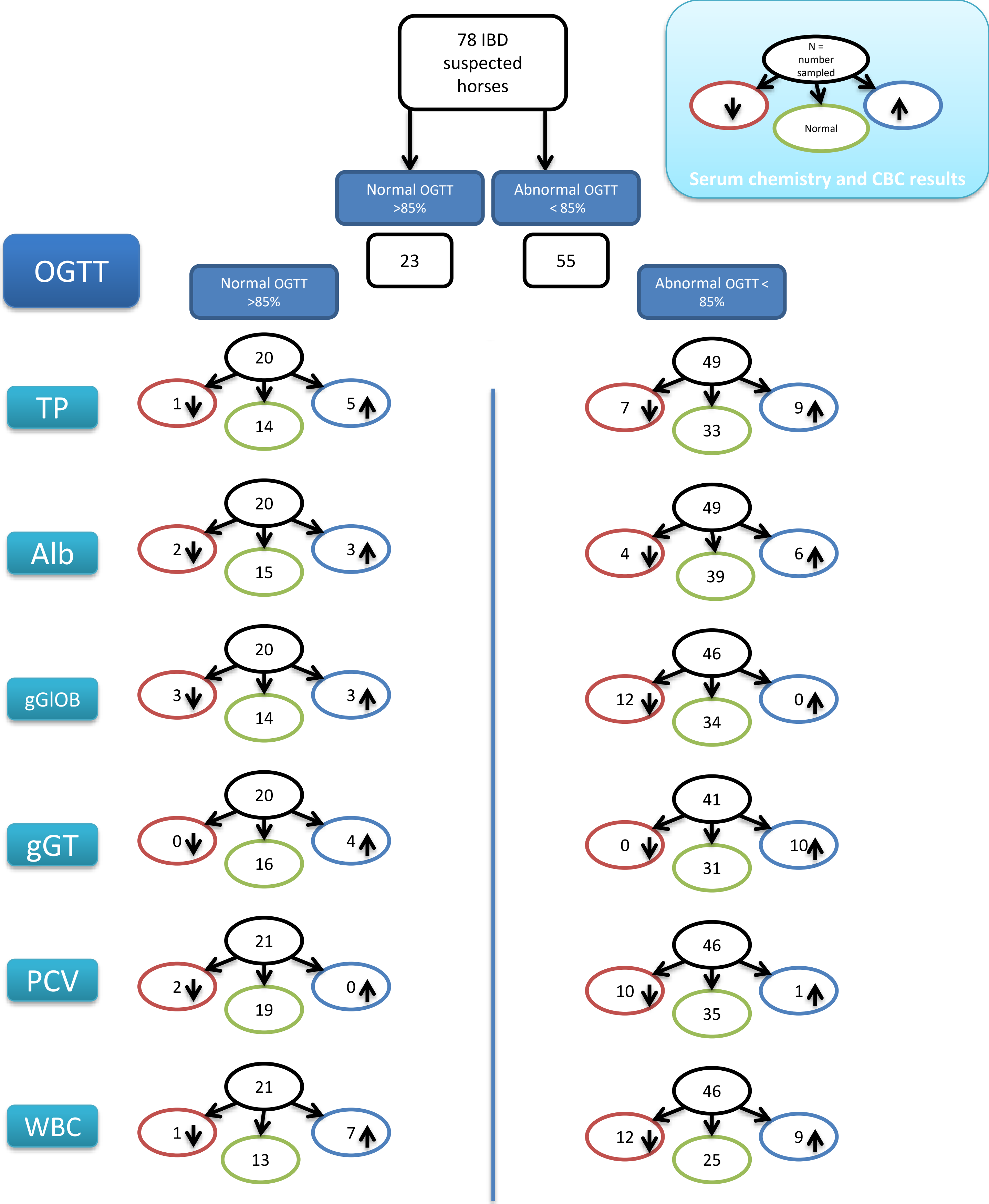

Additional figure 3: Overview of serum chemistry and CBC results of the equine IBD suspected study population, classified by OGTT result (either normal: left panel, or abnormal: right panel), depicted as either below reference ↓, within or above ↑ reference range.
