# Supplementary material for: Inflammatory bowel disease (IBD) in horses: a retrospective study exploring the value of different diagnostic approaches
Source: BMC Vet Res. 2018 Jan 19;14:21. doi: 10.1186/s12917-018-1343-1 (PMC5775604; doi:10.1186/s12917-018-1343-1)
Supplement: Supplementary file 4 — Detailed overview of applied treatments in the study population. (DOCX 14 kb) [file 12917_2018_1343_MOESM4_ESM.docx]

**Additional file 4**: An overview of the applied treatments and the respective number of IBD suspected horses receiving that treatment.

| **Therapy** | **Number of horses** |
| --- | --- |
| Only prednisolone (1 mg/kg BW) | 36 |
| Only dexamethasone (0.06 mg/kg BW) | 0 |
| Only omeprazole (2-4 mg/kg BW) | 7 |
| Prednisolone in combination with dexamethasone | 1 |
| Prednisolone in combination with omeprazole | 19 |
| Dexamethasone in combination with omeprazole | 1 |
| Other | 4 |
| **Total** | **68** |
